# Do Motor Difficulties in Infancy Predict 7-year-olds’ Behavioural Health? Findings from the Avon Longitudinal Study of Parents and Children

**DOI:** 10.1016/j.jpedcp.2025.200167

**Published:** 2025-07-28

**Authors:** Emma Butler, Mary Clarke, Michelle Spirtos

**Affiliations:** 1 Department of Population Health, Royal College of Surgeons Ireland, Dublin, Ireland; 2 Department of Psychology, School of Population Health & Department of Psychiatry, Royal College of Surgeons Ireland, Dublin, Ireland; 3 Department of Occupational Therapy, Trinity College Dublin, Dublin, Ireland

**Keywords:** ALSPAC, child behavioral health, longitudinal, motor skills, prognostic

## Abstract

**Objective:**

To examine whether fine- and gross-motor skills in infancy predict child behavioral health at 7 years of age.

**Study design:**

Longitudinal cohort data from 6,709 English children were analyzed using regression techniques to investigate whether fine- and gross-motor skills at 18 months, measured by Denver developmental categorized age-adjusted Z-scores, predicted behavioral health at 7 years of age, measured by the total-score on the Strengths and Difficulties Questionnaire.

**Results:**

A dose-response relationship exists between fine-motor skills at 18 months and behavioral health at 7 years of age. In fully-adjusted models, (cumulative sociodemographic risk, sex, history of maternal psychological difficulties, gross-motor skills, and gestational age), the odds of experiencing clinical levels of behavioral health symptoms at 7 years of age decreased as fine-motor skills increased. Odds ratios were 0.5 (95% CI 0.2-1.3) for children with below, 0.3 (95% CI 0.1-0.8) for slightly-below, 0.2 (95% CI 0.1-0.4) for average and, 0.1 (95% CI 0.1-0.4) for above-average fine-motor skills compared to children with well-below fine-motor skills. Children with well-below, below, and slightly-below-average fine-motor skills reported almost 6-times, 3-times and double higher rates of clinical behavioural health symptoms at 7-years compared to children with average or above fine-motor-skills. Gross-motor skills were not prognostic of later behavioural health.

**Conclusions:**

Infants with any level of fine-motor difficulties had higher rates of parent-reported behavioural health symptoms at 7-years. Fine-motor but not gross-motor skills in infancy are predictive of poor behavioural health at 7-years. Examining how numerous factors such as motor skills, gestational-age and sociodemographic risk combine to predict risk of poor behavioural health may be more useful than considering any individual predictor in isolation.

Decreased motor skills have been associated with poorer behavioral/mental health in studies involving children who have clinical motor impairments such as Developmental Coordination Disorder or those with other neurodevelopmental disorders 1, 2, 3, 4 Both populations have been identified as experiencing higher rates of poor behavioral/mental health than typically developing peers ^2^^,^^5^, ^6^, ^7^, ^8^ However, focusing on the extreme end of the motor skills continuum ie children with a diagnosed motor disorder, may overestimate the relationship between motor skills and behavioral health in the wider population.

In studies examining this relationship there is inconsistency in which motor skills are measured.^9^ Fine-motor skills involve small precise movements of hands whilst gross-motor skills relate to the use of larger muscle groups to move arms, legs, and the body. Some studies only consider gross-motor skills, ^10^ others focus on fine-motor skills ^1^^,^^4^, some consider both ^11^^,^^12^ and others do not distinguish.^7^^,^^8^^,^^13^^,^^14^ Factors which have been identified to influence the relationship between motor skills and behavioral health include sex ^14^^,^^15^ and birthweight.^15^ Additionally, gestational age correlates with fine- and gross-motor scores in infancy.^12^

In practice, knowing whether motor difficulties are prognostic of later outcomes in other areas is beneficial. Predictors, which may be causal or non-causal in nature, should be clinically or biologically plausible.^16^ Motor skills are plausibly related to behavioral health via direct (cerebellum, basal ganglia, frontal cortex brain function differences) ^17^^,^^18^ or indirect (fine-motor difficulties can lead to lower self-confidence/academic attainment which can lead to poor behavioral health) ^3^ effects. As most studies are cross-sectional in nature, the temporal order of the relationship is difficult to establish. Of the few that utilize a longitudinal design and find a prognostic relationship, they consider motor ability in middle childhood and its relationship to behavioral/mental health in either adolescence ^14^ or adulthood.^13^ In a prediction framework, delaying predicting risk until a child exhibits motor difficulties in middle childhood delays access to early intervention. One study ^12^ considered motor ability in infancy and early childhood (4-months to 4-years) and its relationship to anxiety and depression at 6-12 years, finding the variability in gross-motor scores but not fine-motor associated with later mental health. Limitations of this study were a small sample size (n = 50) and lack of adjustment for socioeconomic status. We have previously demonstrated a dose-response relationship between cumulative sociodemographic risk and child mental health.^19^ It is unknown if a relationship between motor skills and behavioral health exists within the general population, whether motor skills predict later behavioral health after controlling for environmental factors and whether there is specificity in the relationship ie is it fine-motor or gross-motor or both factors driving the relationship with behavioral health. If motor problems are found to precede behavioral health difficulties, this information could be used to identify children most at-risk of later poor behavioral health.

We sought to examine whether motor difficulties (fine- and gross-motor) in infancy predicted the child's behavioral health at 7-years of age. We aimed to establish whether: (1) there is an association between motor skills in infancy and behavioral health symptoms at 7-years of age (2) infant motor skills predict behavioral health at 7-years of age.

## Method

Data were from first-generation children of the Avon Longitudinal Study of Parents and Children (ALSPAC). Pregnant women resident in Avon, UK with expected dates of delivery between 1st April 1991 and 31st December 1992 were invited to participate. ALSPAC initially enrolled 14, 541 pregnancies, from which 14,062 live-births occurred with 13,988 children alive at 1-year-old. Details of the design, sample, and measures presented elsewhere.^20^^,^^21^ Ethical approval for the study was obtained from the ALSPAC ethics and law committee and the local research ethics committees. Informed consent for the use of data collected via questionnaires and clinics was obtained from participants following the recommendations of the ALSPAC ethics and law committee at the time. Please note that the study website contains details of all the data that is available through a fully searchable data dictionary and variable search tool: (https://www.bristol.ac.uk/alspac/researchers/our-data). This study uses data gathered in pregnancy, birth, 18-months, and 7-years-old. This secondary analysis was approved by the research ethics committee for the Royal College of Surgeons Ireland. (RCSI RIMS 212610659).

### Measures

#### Motor Skills at 18-Months-old

The child's fine- and gross-motor development Z-scores at 18-months-old measured by the Denver developmental assessment ^22^ completed by the mother were used. Z-scores were available as constructed age-adjusted variables with a mean of 0 and SD of 1. We also categorized this variable into SD groupings.

#### Maternal History of Psychological Difficulties

This was based on maternal responses on a self-completed questionnaire at ∼12 weeks gestation to questions regarding experiencing drug addiction, alcoholism, schizophrenia, anorexia, severe depression, or ‘other’ psychiatric problems in the past (binary no/yes).

#### Infant Sex

This was self-reported by the mother at ∼ four weeks postnatal (male/female).

#### Cumulative Sociodemographic Risk

Based on previous work ^19^^,^^23^ we created a sociodemographic risk score based on postnatal sociodemographic characteristics of the child's environment at 1-month-old (Table Supp.1; available at www.jpeds.com) that were self-reported by the mother on questionnaires administered between eight-weeks gestation and four-weeks postnatal. It comprised maternal age, race, Townsend-area-level income quintile, maternal education, and relationship status.^23^ Both higher (more than 36-years) and lower maternal age at birth (<26-years) were considered risks. Race (self-reported) was dichotomized as “white” and “other” with “white” considered less risk, as per the original study by Li et al. (2020).^23^ A 3-category variable was created from Townsend income quintiles to denote high (Q4 and 5), middle (Q3), and low-income (Q1 and 2). Maternal education was categorized as (1) no formal education/CSE (2) O-level or vocational, (3) A-level and (4) degree. Lower educational attainment corresponded to higher risk scores. Lastly, having a partner was considered low risk, and not having a partner considered higher risk. Values were then combined and divided into four sociodemographic-risk categories: none, low, moderate, and high.

#### Gestational-Age Group

We categorized gestation, measured as length of pregnancy in weeks as: ‘Term’ (more than 37-weeks), ‘moderately-late preterm’ (more than 32-36^6^ weeks), ‘very preterm' (more than 28-31^6^ weeks) and ‘extremely preterm' (<28-weeks).

#### Behavioral Health

The parent-rated Strengths and Difficulties Questionnaire (SDQ) ^24^ was completed by the mother when the child was 7-years-old as a measure of child behavioral health. The SDQ is a valid and reliable instrument for screening for emotional and behavioral problems in children aged 3-16 years and is widely used in research and clinical practice.^25^ The SDQ contains 25-items on a 3-point Likert scale (0 = not true; 1 = somewhat true; 2 = certainly true), five items are reverse scored. Item scores are aggregated into 5 subscales. The first four subscales combine to calculate a total-score ranging from 0 to 40. Parent-reported SDQ-total scale has higher internal consistency (Cronbach's-alpha = 0.82) and test-retest reliability than the four subscales.^26^ Higher scores indicate higher difficulties. The SDQ-total was also dichotomized at the recommended cut-off of 17 or above representing children experiencing symptomology in the clinical range (sdqinfo.org).

### Statistical Analysis

Analysis was conducted using STATA v.17.^27^ To determine whether there was a higher occurrence of clinical behavioral health problems among children with motor difficulties in infancy, chi-square tests were conducted. Tests to determine whether the total-SDQ score was normally distributed were carried out. An analysis of variance test was used to determine if there was a difference in mean SDQ-total score between motor groups. Adjustments were made for multiple corrections and unequal variances between groups (Dunn's test with Bonferroni correction after Kruskal-Wallis). Logistic regression analysis was used to establish whether motor group independently predicted clinical levels of behavioral health symptoms. Multiple regressions adjusting for sex, sociodemographic risk, gestational age-group, motor abilities, and history of maternal mental health difficulties was completed.

### Participants Included in Analysis

Of the original 14,633 babies, 8,351 took part at 7-years and had the total-SDQ score available. 6,709 who participated at 7-years had all covariates of interest.

### Missing Data

We compared characteristics of participants included in our analysis n = 6709 to those excluded due to withdrawal, missing outcome or missing independent variable data n = 8,351 on all variables of interest (Table Supp.2; available at www.jpeds.com).

## Results

Excluded individuals experienced higher levels of sociodemographic risk, history of maternal psychological difficulties, preterm births, and total-SDQ score (Table Supp.2).

### Fine-Motor

Distribution characteristics by fine-motor group are presented in Table.I. At 18-months, 71.1% had fine-motor skills in the average range, 14.3% in the above-average range, 11.7% were slightly below-average (−1 to −2SD), 2.4% quite below-average (−2 to −3SD) with 0.5% significantly below-average (≤-3SD). Males were more likely than females to have lower fine-motor skills (x^2^ = 32.9, *P* = <.001, Cramers V = 0.07). There was no difference between the fine-motor groups regarding cumulative sociodemographic risk. Children with the worst fine-motor skills (≤-3SD) were significantly more likely to have a mother with a history of psychological difficulties (x^2^ = 13.7, *P* = .008, Cramers V = 0.05). Children with any level of prematurity had higher rates of ‘slightly below-average’ fine-motor skills. Children with any level of fine-motor difficulties had higher rates of poor gross-motor skills.

**Table I:** Distribution Characteristics by Fine-Motor Group in Infancy

	Total (n = 6709)	∗Well-below (n = 31 0.5%)	Below (n = 164 2.4%)	Slightly-below (n = 785 11.7%)	Average (n = 4773 71.1%)	Above-average (n = 956 14.3%)
Sex (% male)	51.1	74.2	59.2	55.5	51.2	44.7
Cumulative sociodemographic risk (%)						
None	20.3	ˆ	17.7	19.1	20.6	20.2
Low	48.8	51.6	48.8	47.5	48.9	49.0
Moderate	28.1	29.0	26.8	29.4	27.8	28.5
High	2.9	ˆ	6.7	4.0	2.6	2.4
History of maternal psychological difficulties (% yes)						
	10.3	25.8	6.1	11.3	10.4	9.1
Gestational-age (med, IQR)	40 (39-41)	39 (38-41)	39 (38-40)	40 (38-40)	40 (39-41)	40 (39-41)
Preterm birth category (%)						
Term	95.4	93.6	92.7	91.3	95.9	96.6
MLPT	4.1	ˆ	4.9	7.3	3.8	3.2
VPT	0.4	ˆ	ˆ	1.4	0.2	ˆ
EPT	0.1	ˆ	ˆ	ˆ	ˆ	ˆ
Gross-motor Z-score (med, IQR)	.20 (−0.5 to 0.8)	−1.90 (−3.3 to −0.6)	−0.52 (−1.6 to −0.2)	−0.16 (−0.9 to 0.5)	.20 (−0.5 to 0.8)	.55 (−0.1 to 0.9)
Gross-motor category (%)						
<=−3 SD	1.4	25.8	7.9	2.2	1.1	ˆ
−3 to −2 SD	1.5	ˆ	7.9	3.1	1.1	0.6
−2 to −1 SD	8.8	22.6	23.2	14.5	8.1	4.5
−1 to +1 SD	88.4	38.7	61.0	80.3	89.8	94.4
Clinical SDQ (%yes)	5.2	25.8	12.8	8.7	4.5	3.7
SDQ-categories (%)						
Close to average	89.4	64.5	79.9	82.8	90.5	92.3
Slightly raised	5.4	ˆ	7.3	8.5	5.1	4.1
High	3.1	ˆ	7.3	4.8	2.7	2.4
Very high	2.1	ˆ	5.5	3.8	1.8	1.3
SDQ-total (M, SD)	7.4 (4.7)	12.3 (5.5)	9.3 (5.5)	8.6 (5.2)	7.3 (4.6)	6.7 (4.5)
Min-max	0-31	1-25	1-28	0-31	0-31	0-27

*EPT*, extremely preterm; *Med*, median; *MLPT*, moderately late preterm; *VPT*, very preterm; *SDQ*, Strengths and Difficulties Questionnaire.

∗ Well-below (<=−3SD), below (−3 to −2SD), slightly below (−2 to −1 SD), average (−1 to +1SD), above-average (+1 to +2SD). ˆcell count 5 or less and cannot be specified as per request of ALSPAC committee.

A one-way analysis of variance examined the effect of fine-motor group at 18-months on SDQ-total at 7-years F(4, 6704) = 33.79, *P* = <.001. As the variances between the groups were unequal (Bartletts equal-variance test: chi^2^ ^4^ = 42.28, *P* < .001) we reran the analysis using the Kruskal-Wallis nonparametric test. This confirmed a significant difference in ranks between the fine-motor groups, chi^2^ ^4^ = 109.14, *P* < .001. There was a dose-response relationship between level of fine-motor skills and mean SDQ-total score at 7-years. As fine-motor skills decreased, mean SDQ-total score increased. SDQ-total mean 12.3, 9.3, 8.6, 7.3, 6.7 for ≤ −3SD, −3 to −2SD, −2 to -−1SD, −1 to +1SD, and +1 to +2SD, respectively. After applying the Bonferroni correction for multiple comparisons, a significant difference remained in total-SDQ score between all fine-motor groups except between −3 to −2 SD and −2 to −1SD (Table I). This explained 2.0% of the variance in the total-SDQ score with a small effect size: eta^2^ = 0.02 (95% CI 0.01-0.03).

This dose-response pattern was also evident when using the dichotomized SDQ variable as the outcome. Children with any level of fine-motor difficulties (all groups < -1SD) reported much higher rates (almost 6 times higher, 3 times higher, and double for ≤ -3SD, −3 to −2 and −2 to -1SD, respectively compared to the average-range group −1 to +1SD) of children categorized as experiencing clinical levels of behavioral health symptoms at 7-years. Pearson chi^2^ ^4^ = 83.7, *P* = <.001, Cramers V = 0.10. This was not merely due to higher rates in the most extreme SDQ-category but chi-square analyses identified a significant difference between fine-motor groups in all SDQ-groups: x^2^ = 112.4, *P* = <.00, Cramers V = 0.07 (Figure).

**Fig:**
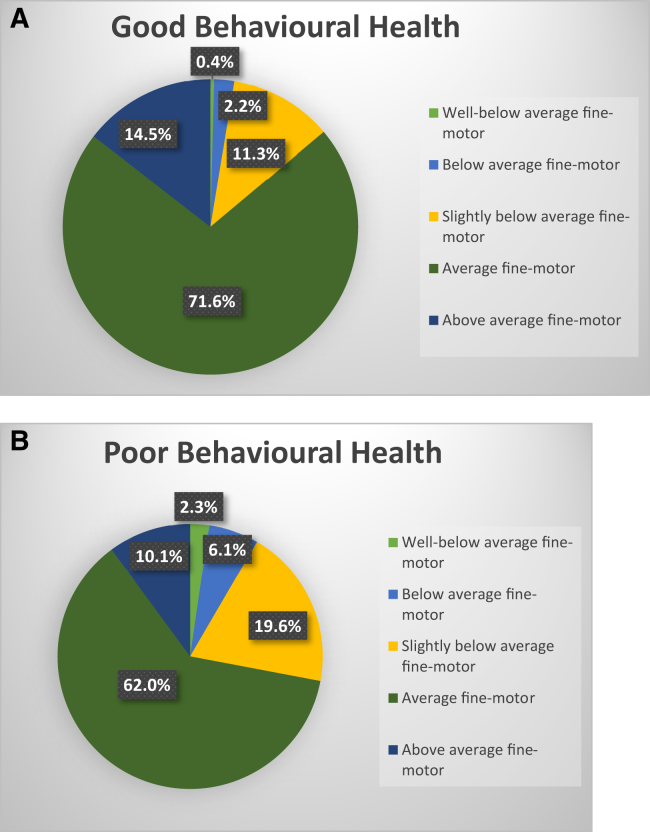
Proportion of children with non-clinical **A,** and clinical **B,** behavioral health symptoms at 7-years of age by the fine-motor group in infancy.

### Gross-Motor

No child displayed above-average gross-motor skills at 18-months (Table Supp.3; available at www.jpeds.com). A total of 88.4% had average gross-motor skills, 8.8% were slightly below-average (−1 to −2SD), 1.5% quite below-average (−2 to −3SD) with 1.4% significantly below-average (≤-3SD). Children with below-average gross-motor skills had higher rates of behavioral health symptoms at 7-years than children with average gross-motor skills (x^2^ = 8.7, *P* = .03, but with negligible effect Cramers V = 0.03). This was driven by the children with the worst gross-motor skills (≤-3SD). The significant difference in SDQ-total score between ≤ -3SD group and the average group (−1 to +1SD) did not survive adjustment for multiple corrections. There was no difference in gross-motor skills by sex but children with no cumulative sociodemographic risk were more likely to have slightly below- and quite below-average gross-motor skills. (x^2^ = 40.0, *P* = <.001, Cramers V = 0.05). There was no significant difference in gross-motor skills in relation to maternal history of psychological difficulties (x^2^ = 3.43, *P* = .33, Cramers V = 0.02). Children with any level of gross-motor difficulty had higher rates of fine-motor difficulties. Children with any level of prematurity had significantly higher rates of all levels of below-average gross-motor skills in a dose-response fashion (Table Supp.3; available at www.jpeds.com).

To explore whether the difference in SDQ-score by fine-motor groups is clinically meaningful we considered the proportion of fine-motor groups in the clinical and nonclinical SDQ range (Figure) There are much higher rates of all below-average fine-motor groups in the clinical range group and a lower proportion of children from the average and above-average fine-motor groups in the clinical range at 7-years of age compared to the non-clinical groups (Figure). When considered by gross-motor groups (Fig Supp.1; available at www.jpeds.com) it confirmed that there is no difference in clinical-outcome proportions by gross-motor groups.

The logistic model in Table.II shows that fine-motor skills (model 1a), but not gross-motor skills (model 1b) are a significant predictor of the odds of experiencing later poor behavioral health. Increasing fine-motor skills are associated with decreasing odds of experiencing later behavioral health difficulties in a dose-response fashion.

**Table II:** Logistic Regression Models for Clinical Behavioral Health Symptoms at 7 Years of Age

(n = 6709)	OR	Std. error	z	P > z	95% CI	% Change
Model 1a (fine-motor only)						
<=−3SD base						
−3 to −2SD	0.42	.20	−1.83	0.07	0.17-1.07	
−2 to −1SD	0.27	.12	−3.02	0.002	0.12-0.63	
−1 to +1 SD	0.14	.06	−4.80	0.00	0.06-0.31	
+1 to +2 SD	0.11	.05	−4.97	0.00	0.05-0.26	
Model 1b (gross-motor only)						
<=−3SD base						
−3 to −2SD	0.58	.34	−0.91	.36	.18-1.86	
−2 to −1SD	0.83	.33	−0.48	.63	.38-1.82	
+1 to +2 SD	0.56	.21	−1.56	.27	.27-1.16	
Model 2 (fully-adjusted model)						
Fine-motor (<=−3SD base)						
−3 to −2SD	0.50	.25	−1.40	.16	0.19-1.32	−50.0
−2 to −1SD	0.33	.15	−2.45	0.01	0.13-0.80	−67.4
−1 to +1 SD	0.17	.08	−3.95	0.00	0.07-0.41	−83.0
+1 to +2 SD	0.14	.07	−4.07	0.00	0.06-0.37	−85.6
Sex–male base						
Female	0.71	.08	−2.91	0.004	0.57-0.90	−28.3
Sociodemographic risk – none base						
Low	1.83	.34	3.31	0.001	1.28-2.63	83.7
Moderate	2.22	.43	4.15	0.00	1.52-3.24	122.1
High	3.76	1.08	4.59	0.00	2.14-6.61	275.9
History of maternal psychological difficulties–no base						
Yes	2.22	0.32	5.59	0.00	1.68-2.93	121.9
Gross-motor (<=−3SD base)						
−3 to −2SD	0.65	.40	−0.70	.49	0.20-2.17	−34.7
−2 to −1SD	1.17	.51	0.37	.72	.50-2.75	17.2
−1 to +1 SD	0.89	.37	−0.28	.78	.40-2.01	−10.9
Preterm birth category (term base)						
MLPT	1.30	.32	1.08	.28	.81-2.10	30.1
VPT	0.57	.60	−0.53	.60	.07-4.45	−42.6
EPT	2.35	2.65	0.76	.45	.26-21.44	135.1

*EPT*, extremely preterm; *MLPT*, moderately late preterm; *VPT*, very preterm.

% change–this is the percent change in the odds of the outcome (having clinical behavioral health symptoms) for a one-unit increase in the predictor holding all other variables constant. It can be used to support interpretation instead of interpreting the multiplicative or factor change in the outcome.

In the fully adjusted model (model 2), adjusted for sex, sociodemographic risk, history of maternal psychological difficulties, gross-motor skills, and preterm birth category, the odds of experiencing later behavioral health difficulties based on fine-motor category in infancy remains largely unchanged, thereby highlighting its potential as a predictor of behavioral health over and above other known predictors. We calculated the predicted probability of having parent-reported behavioral health symptoms at 7-years of age for each fine-motor group which highlighted that there is a dose-response relationship with increased risk evident for all below-average fine-motor groups.

Sex, sociodemographic risk, and maternal history of psychological difficulties all have a significant relationship with the odds of experiencing later poor behavioral health in addition to fine-motor skills. Examining how numerous factors such as motor skills, gestational-age, and sociodemographic risk combine to predict risk of poor behavioral health may be more useful in practice than considering any individual predictor in isolation.

## Discussion

We investigated whether motor skills at 18-months predicted the child's behavioral health at 7-years of age. Most children in all fine-motor groups had no behavioral health symptoms, however we found that children with any level of below average fine-motor skills had significantly higher rates of poor behavioral health symptoms at 7-years of age. Below-average fine-motor skills at 18-months remained a significant predictor of behavioral health at 7-years of age in a dose-response fashion in an adjusted-model including child sex, cumulative sociodemographic risk, history of maternal psychological difficulties, gross-motor skills, and preterm birth.

Previous studies concur with our findings, supporting a relationship between manual coordination and SDQ-total in 4-11-year-olds ^1^ and fine-motor skills (DCDQ-J) and SDQ-total in 5-6-year-olds.^4^ Our study significantly furthers this knowledge by showing that these fine-motor difficulties precede the behavioral health difficulties and can be considered prognostic of later behavioral health in a very large sample. Contrarily, a study ^28^ found that motor scores (a composite of fine- and gross-motor measured using the MABC-2) at 3 years did not predict SDQ-score at 5 years, however they themselves noted that they did not have adequate power to conclude the absence of a relationship (n = 38). Furthermore, they did not examine fine- and gross-motor separately.

Using this population-based cohort we provided evidence that the association between motor skills and behavioral health applies to all children, not just to clinical samples of children with significant, diagnosed impairments of motor skills. This is supported in another study ^13^ of normal birthweight children; for each one-point decrease in total-motor score (a composite score of fine- and gross-motor as measured using the BOT-SF) at 8-years of age, the odds of major depressive disorder at 29-36 years increased by 10%. Our study adds to this evidence by showing that even though fine- and gross-motor skills are related, children with fine-motor difficulties have higher rates of gross-motor difficulties and vice versa, it is in fact the fine-motor skills rather than the gross-motor skills that are driving the association with later behavioral health. This shows a specificity in the relationship between fine-motor skills and behavioral health outcomes.

Although a minority of children in each fine-motor group had poor behavioral health, we found that children with any level of below-average fine-motor skills had significantly worse behavioral health at 7-years of age than the infants who had average or above-average fine-motor skills indicating that fine-motor difficulties should not be considered as a dichotomous risk variable with an arbitrary cut-off for risk of behavioral health, but rather viewed on a continuous scale as risk decreases with each fine-motor SD increase. A large cross-sectional study ^29^ supports understanding motor skills as a continuous rather than a dichotomous construct. Yet in practice, access to interventions for fine-motor skills are typically dependent on an arbitrary cut-off, usually the 5th percentile, whilst this study illuminates that children, up to and including the 16th percentile also have significantly increased risk. Danks et al. (2022) support the prognostic value of ‘low-normal’ motor skills, (as measured by the neurosensory motor developmental assessment), for later development, recommending referral of these infants to early intervention.^30^

The significant relationship between motor skills and behavioral health difficulties continues to be under-recognized. A dose-response relationship between fine-motor skills has previously been identified with later psychosis ^31^, we demonstrated this with behavioral health more broadly.^32^ Screening for associated later behavioral health problems in children who are experiencing motor difficulties is indicated by the evidence shown here. Furthermore, in infancy, a simple, brief, direct assessment and/or parent-report of fine motor development may be flagged by a primary care physician as a proxy for broader developmental concerns and/or need for continued monitoring of child's development over time. Considering the contribution of numerous factors when predicting-risk of behavioral health would arguably increase the accuracy of the prediction than considering any one factor in isolation such as fine-motor skills or gestational-age. Developing clinical risk-prediction algorithms may be more useful in clinical practice than searching for singular factors.

### Limitations

The use of a large, population-based prospective longitudinal sample is a significant strength of this study. We did not have information available as to whether the child had a specific mental health diagnosis and could not examine whether significant motor difficulties were confined to diagnostic groups. However transdiagnostic approaches which focus on symptoms and soften or remove the boundaries between traditional categorical disorders are increasingly adopted in research and practice ^33^ and the SDQ has been found to be a valuable screening tool for identifying behavioral health difficulties in children who are struggling regardless of whether they are considered to be typically developing or having a specific diagnosis.^33^^,^^34^ We also did not have a measure of their intelligence at 18-months. However, we ran the analysis adjusting for the social and communication domains of development at 18-months (available from author) and the findings did not meaningfully change. The most vulnerable children were lost to attrition and the findings may not be generalizable to that group. ALSPAC is a predominantly White cohort which again could impact generalizability. Furthermore, due to small cell sizes in extremely and very-preterm groups, we may not have had adequate power to identify more significant differences in these groups. Lastly motor difficulties were based on parent-report, not clinical observations, which could impact the validity, however parent-report of motor skills has been found to be reliable and dependable in comparison with direct assessment.^35^^,^^36^ Furthermore, children who later were reported as having a dyspraxia diagnosis at 9-years had lower fine- and gross-motor Z-scores in infancy (available from author) supporting the validity of the motor Z-scores used here.

## Conclusion

Infants experiencing fine-motor difficulties have significantly higher rates of behavioral health symptoms at 7-years of age. Fine-motor problems in infancy are prognostic of later behavioral health and thus could be considered a marker for later behavioral health risk. As a result, in practice, screening of fine-motor skills should not be overlooked. Children with below-average fine-motor skills, regardless of their gross-motor abilities should be referred for early intervention.

## Data Statement

Data sharing statement available at www.jpeds.com.

## CRediT authorship contribution statement

**Emma Butler:** Writing – review & editing, Writing – original draft, Visualization, Software, Funding acquisition, Formal analysis, Conceptualization. **Mary Clarke:** Writing – review & editing, Supervision, Conceptualization. **Michelle Spirtos:** Writing – review & editing, Supervision, Conceptualization.

## Declaration of Competing Interest

This project was funded by the Health Research Board Ireland SPHeRE-2018-1. The funders had no role in any aspect of this study. The UK Medical Research Council and Wellcome (grant reference: 217065/Z/19/Z) and the University of Bristol provide core support for ALSPAC. A comprehensive list of grants funding is available on the ALSPAC website (http://www.bristol.ac.uk/alspac/external/documents/grant-acknowledgements.pdf). This publication is the work of the authors and E.B. will serve as guarantor for the contents of this paper. E.B. reports financial support was provided by Health Research Board

## Supplementary Data

Supplementary Data 2

Data Statement
