# Supplementary material for: Do Motor Difficulties in Infancy Predict 7-year-olds’ Behavioural Health? Findings from the Avon Longitudinal Study of Parents and Children
Source: J Pediatr Clin Pract. 2025 Jul 28;17:200167. doi: 10.1016/j.jpedcp.2025.200167 (PMC12356018; doi:10.1016/j.jpedcp.2025.200167)

Table Supp.1: distribution (%) of individual factors comprising the cumulative sociodemographic risk score.

| N=10378 | 0 no risk | 1 low risk | 2 moderate risk | 3 highest risk |
| --- | --- | --- | --- | --- |
| Maternal Race | 97.7 | 2.3 |  |  |
| Maternal age | 66.0 | 34.0 |  |  |
| Maternal relationship | 97.8 | 2.2 |  |  |
| Maternal education | 13.6 | 23.4 | 45.0 | 18.0 |
| Family income quintile | 42.7 | 19.6 | 37.8 |  |
| Cumulative sociodemographic risk | 16.8 | 45.8 | 32.0 | 5.4 |

Note: this was constructed for all participants who had the 5 individual variables available not just those included in the analytic sample.

Table Supp.2: comparing participant characteristics between those included and those excluded.

|  | Total N=14633 | Included N=6709 | Excluded N=7924 |
| --- | --- | --- | --- |
| Sex (%male)N=14118 | 51.7 | 51.1 | 52.2 |
| Sociodemographic risk (%): N=10378 | | | |
| None | 16.8 | 20.3 | 10.3 |
| Low | 45.8 | 48.8 | 40.3 |
| Mod | 32.0 | 28.1 | 39.2 |
| High | 5.4 | 2.9 | 10.2 |
| Maternal history of psychological difficulties (%yes)  N=12548 | 12.0 | 10.3 | 14.0 |
| Gestational age (med, IQR)  N=14063 | 40 (39-41) | 40 (39-41) | 40 (38-41) |
| Birth category (%): N=14063 | | | |
| Term 37+ | 93.5 | 95.4 | 91.9 |
| MLPT 32-36+6 | 5.3 | 4.1 | 6.4 |
| VPT 28-31+6 | 0.8 | 0.4 | 1.1 |
| EPT <28 | 0.4 | 0.1 | 0.6 |
| Fine Motor Z-score (M, SD): N=10503 | 0.00 (1.0) | 0.02(.96) | -0.04 (1.1) |
| Fine Motor category (%): N=10503 | | | |
| <= -3SD | 0.7 | 0.5 | 1.2 |
| Between -3 and -2 SD | 2.6 | 2.4 | 2.8 |
| Between -2 and -1 SD | 11.8 | 11.7 | 11.9 |
| Between -1 and +1 SD | 70.6 | 71.1 | 69.7 |
| Between +1 and +2SD | 14.3 | 14.3 | 14.4 |
| Gross Motor Z-score (med, IQR): N=10551 | 0.20 (-0.5-0.8) | 0.20 (-0.5-0.8) | 0.20 (-0.5-0.8) |
| Gross Motor category (%): N=10551 | | | |
| <= -3SD | 1.6 | 1.4 | 1.9 |
| Between -3 and -2 SD | 1.5 | 1.5 | 1.7 |
| Between -2 and -1 SD | 8.2 | 8.8 | 7.1 |
| Between -1 and +1 SD | 88.7 | 88.4 | 89.3 |
| ClinicalSDQ (% yes): | 5.6 | 5.2 | 7.4 |
| SDQ-categories (%): | | | |
| Average | 88.6 | 89.4 | 85.3 |
| Slightly Raised | 5.8 | 5.4 | 7.3 |
| High | 3.3 | 3.1 | 4.0 |
| Very High | 2.4 | 2.1 | 3.4 |
| SDQ-total (M, SD) | 7.6 (4.9) | 7.4 (4.7) | 8.1 (5.2) |

MLPT – moderately late preterm. VPT – very preterm. EPT – extremely preterm. Med – Median. IQR – interquartile range. SDQ -strengths and difficulties questionnaire. SD – standard deviation.

Table Supp.3: Distribution characteristics by GROSS-MOTOR groupings in infancy.

|  | Total 7-y  (n=6709) | <= -3SD  (n=94  1.4%) | -3 to -2 SD  (n=97  1.5%) | -2 to -1SD  (n=589  8.8%) | -1-+1SD  (n=5929  88.4%) |
| --- | --- | --- | --- | --- | --- |
| Sex (% male) | 51.1 | 46.8 | 52.6 | 45.5 | 51.7 |
| Cumulative sociodemographic risk (%): | | | | | |
| none | 20.3 | 23.4 | 29.9 | 25.0 | 19.6 |
| low | 48.8 | 55.3 | 49.5 | 52.0 | 48.3 |
| mod | 28.1 | 19.2 | 16.5 | 19.9 | 29.2 |
| High | 2.9 | ^ | ^ | 3.2 | 2.8 |
| History of maternal psychological difficulties (% yes) | 10.3 | 14.9 | 8.3 | 11.4 | 10.2 |
| Gestational Age (med, IQR) | 40 (39-41) | 39 (38-40) | 39 (38-41) | 40 (39-41) | 40 (39-41) |
| Birth category (%): | | | | | |
| Term | 95.4 | 80.9 | 85.6 | 94.9 | 95.8 |
| MLPT | 4.1 | 9.6 | 9.3 | 3.9 | 4.0 |
| VPT | 0.4 | 7.5 | ^ | 1.0 | 0.1 |
| EPT | 0.1 | ^ | ^ | ^ | ^ |
| Fine Motor Z-score (med, IQR) | .25 (-0.6-0.7) | -0.70(-1.9-0.0) | -0.58(-1.6-0.2) | -0.24(-1.2-0.4) | .35(-0.4-0.8) |
| Fine-Motor category (%): | | | | | |
| <=-3 | 0.5 | 8.5 | ^ | 1.2 | 0.2 |
| -3 to -2 | 2.4 | 13.8 | 13.4 | 6.5 | 1.7 |
| -2 to -1 | 11.7 | 18.1 | 24.7 | 19.4 | 10.6 |
| -1 to +1 | 71.1 | 54.3 | 51.6 | 65.7 | 72.3 |
| +1 to +2 | 14.3 | ^ | 6.2 | 7.3 | 15.2 |
| Clinical SDQ (%yes) | 5.2 | 8.5 | ^ | 7.1 | 4.9 |
| SDQ categories (%): | | | | | |
| Close to average | 89.4 | 83.0 | 90.7 | 87.4 | 89.7 |
| Slightly raised | 5.4 | 8.5 | ^ | 5.4 | 5.4 |
| High | 3.1 | ^ | ^ | 4.1 | 3.0 |
| Very high | 2.1 | ^ | ^ | 3.1 | 1.9 |
| SDQ-total (M, SD)  Min-max | 7.4 (4.7)  0-31 | 8.8 (5.2)  1-24 | 7.7 (4.7)  1-22 | 7.9 (5.1)  0-28 | 7.4 (4.7)  0-31 |

^cell count 5 or less. MLPT – moderately late preterm. VPT – very preterm. EPT – extremely preterm. Med – Median. IQR – interquartile range. SDQ -strengths and difficulties questionnaire. SD – standard deviation.

Fig Supp.1: proportion of children with non-clinical and clinical behavioural health at 7-years by gross-motor Z-score group at 18-months is not different.


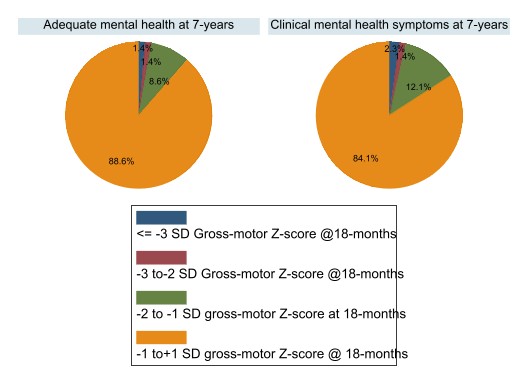

Supplement: Supplementary Data 1 [file mmc1.docx]
