# Supplementary material for: Do Motor Difficulties in Infancy Predict 7-year-olds’ Behavioural Health? Findings from the Avon Longitudinal Study of Parents and Children
Source: J Pediatr Clin Pract. 2025 Jul 28;17:200167. doi: 10.1016/j.jpedcp.2025.200167 (PMC12356018; doi:10.1016/j.jpedcp.2025.200167)
Supplement: Supplementary Data 2 [file mmc2.pdf]

Fine motor questions at 18-months:

She can hold a rattle

She can focus eyes on a small object

She can pick up a small object eg raisin

She can pass object from one hand to another

She can bang together two similar objects that she is holding

She grabs object using whole hand

She can pick up object using finger and thumb only

She will use a pencil to scribble

She can build a tower putting one object on top of another

She can build a tower of 3 blocks

She can build a tower of 4 blocks

She can build a tower of 8 blocks

She can copy a vertical line with a pencil

She points to what she wants

She will turn pages of a book

Gross motor questions at 18-months:

She can stand without being supported for a short period of time

From standing she can bend down and return to standing

She can stand alone for at least one minute without holding on

She can walk while holding someones hand

She can walk alone for at least 5 steps

She can walk backwards 5 steps

She can move around by shuffling on her bottom

She can kick a ball

She can throw a ball

She can balance on one foot for at least one second

She can jump up and down

She can climb stairs

Possible responses are: Can do well, Only done 1-2 times, Not yet started

Spearman correlations between the four Denver developmental domains at 18-months:

```
. spearman fm18 gm18

 Number of obs =   10475
Spearman's rho =       0.2573

Test of H0: fm18 and gm18 are independent
    Prob > |t| =       0.0000

. spearman fm18 comm18

 Number of obs =   10469
Spearman's rho =       0.3027

Test of H0: fm18 and comm18 are independent
    Prob > |t| =       0.0000

. spearman fm18 soc18

 Number of obs =   10495
Spearman's rho =       0.3512

Test of H0: fm18 and soc18 are independent
    Prob > |t| =       0.0000
```

Logistic regression models for having clinical mental health symptoms at 7-years but controlling for social and communication skills at 18-months (kd681 and kd683):

Model 2 (fully-adjusted model) n=6699					
	Odds Ratio	Std. Error	z	P>z	95%Confidence Interval
Fine-motor (<=-3SD base)					
-3 to -2SD	0.56	.28	-1.16	.25	0.21-1.49
-2 to -1SD	0.40	.18	-2.00	0.05	0.16-0.98
-1 to +1 SD	0.23	.10	-3.24	0.00	0.09-0.55
+1 to +2 SD	0.21	.11	-3.13	0.00	0.08-0.56
Sex – male base					
Female	0.80	.09	-1.92	0.06	0.63-1.00
Sociodemographic risk – none base					
Low	1.84	.34	3.34	0.001	1.29-2.65
Moderate	2.31	.45	4.36	0.00	1.59-3.37
High	3.80	1.10	4.62	0.00	2.16-6.69
History of maternal psychological difficulties – no base					
Yes	2.22	0.32	5.57	0.00	1.68-2.93
Gross-motor (<=-3SD base)					
-3 to -2SD	0.65	.40	-0.71	.48	0.19-2.15
-2 to -1SD	1.28	.56	0.57	.57	.54-3.02
-1 to +1 SD	1.11	.47	0.24	.81	.48-2.52
Pre-term birth category (term base)					
MLPT	1.22	.30	0.81	.42	.75-1.98
VPT	0.53	.55	-0.61	.54	.07-4.12
EPT	1.82	2.05	0.53	.60	.20-16.68

Denver social score prorated @ 18-months	0.90	.06	-1.56	.12	.79-1.03
Denver communication score prorated @ 18-months	0.85	.06	-2.40	.02	.75-.97

MLPT – moderately late preterm. VPT – very preterm. EPT – extremely preterm. SD – standard deviation. % change – this is the percent change in the odds of the outcome (having clinical mental health symptoms) for a one-unit increase in the predictor holding all other variables constant. It can be used instead to support interpretation instead of interpreting the multiplicative or factor change in the outcome.

Supporting the validity of the motor Z-scores: ALSPAC 'KU' file – at 9 years – Q: Have you ever been told that your child has dyspraxia?

	Not dyspraxic at 9-years (n=6805)	Dyspraxic at 9-years (n=109)	Missing response to dyspraxic at 9-years (n=3589)
Median (IQR) fine-motor z-score @ 18-months	0.25 (-0.55-0.71)	-0.24 (-1.44-0.43)	0.13 (-0.58-0.71)
Fine-motor Z-score range	-2.62-1.79	-3.91-1.79	-3.08-1.79
Median (IQR) gross-motor z-score @ 18-months	0.20 (-0.52-0.79)	-0.28 (-1.23-0.55)	0.20 (-0.52-0.83)
Gross-motor Z-score range	-3.37-0.95	-5.55-0.95	-5.16-0.95
NB: z-scores have mean of 0 and standard deviation of 1.			

As can be seen from the table above, those who later reported being told the child has dyspraxia at 9-years had significantly lower fine and gross-motor Z-scores when they were 18-months compared to the children who did not report dyspraxia at 9-years. The dyspraxic groups 18-month Z-scores were on average half a standard deviation lower. The children who were missing responses to the dyspraxic question at 9-years had fine and gross-motor Z-scores in line with the group of children who were not reported to be dyspraxic.
