# Supplementary material for: Do Motor Difficulties in Infancy Predict 7-year-olds’ Behavioural Health? Findings from the Avon Longitudinal Study of Parents and Children
Source: J Pediatr Clin Pract. 2025 Jul 28;17:200167. doi: 10.1016/j.jpedcp.2025.200167 (PMC12356018; doi:10.1016/j.jpedcp.2025.200167)
Supplement: Data Statement [file mmc3.docx]

Data Statement

Data is available upon request from https://www.bristol.ac.uk/alspac/researchers/our-data/.
